# Intravenous Enzyme Replacement Therapy in Mucopolysaccharidoses: Clinical Effectiveness and Limitations

**DOI:** 10.3390/ijms21082975

**Published:** 2020-04-23

**Authors:** Rossella Parini, Federica Deodato

**Affiliations:** 1 UOS Malattie Metaboliche Rare, Clinica Pediatrica dell’Università Milano Bicocca, Fondazione MBBM, ATS Monza e Brianza, 20900 Monza, Italy; 2 Division of Metabolic Disease, Bambino Gesù Children’s Hospital, IRCCS, 00165 Rome, Italy

**Keywords:** enzyme replacement therapy, ERT, mucopolysaccharidosis, mucopolysaccharidoses, MPS, laronidase, idursulfase, elosulfase, galsulfase vestronidase

## Abstract

The aim of this review is to summarize the evidence on efficacy, effectiveness and safety of intravenous enzyme replacement therapy (ERT) available for mucopolysaccharidoses (MPSs) I, II, IVA, VI and VII, gained in phase III clinical trials and in observational post-approval studies. Post-marketing data are sometimes conflicting or controversial, possibly depending on disease severity, differently involved organs, age at starting treatment, and development of anti-drug antibodies (ADAs). There is general agreement that ERT is effective in reducing urinary glycosaminoglycans and liver and spleen volume, while heart and joints outcomes are variable in different studies. Effectiveness on cardiac valves, trachea and bronchi, hearing and eyes is definitely poor, probably due to limited penetration in the specific tissues. ERT does not cross the blood–brain barrier, with the consequence that the central nervous system is not cured by intravenously injected ERT. All patients develop ADAs but their role in ERT tolerance and effectiveness has not been well defined yet. Lack of reliable biomarkers contributes to the uncertainties about effectiveness. The data obtained from affected siblings strongly indicates the need of neonatal screening for treatable MPSs. Currently, other treatments are under evaluation and will surely help improve the prognosis of MPS patients.

## 1. Introduction

Mucopolysaccharidoses (MPSs) are a group of inherited, multisystem, lysosomal storage disorders (LSDs) due to defects in glycosaminoglycans (GAGs) degradation, with an overall incidence of 1:20,000 live births [1] (Table 1). The most typical clinical phenotypes of MPSs were recognized as clinical entities at the beginning of the 20th century, but the enzyme defects and the molecular bases were identified only in the second half of the century [2].

The understanding of the pathophysiological mechanisms opened the way to the search for an etiologic treatment. Hematopoietic stem cell transplantation (HSCT) was the first treatment, applied with success to the most severe form of mucopolysaccharidosis (MPS) type I (Hurler) [3]. It was also employed in smaller numbers of patients with MPS II, III, IV and VI with controversial results [4,5,6,7,8,9,10,11,12,13]. Recent evidence suggests HSCT as an acceptable treatment option for MPS II [12,13,14]. Enzyme replacement therapy (ERT) is the other more recent available treatment, which is obtained through recombinant DNA technology. The enzyme, administered weekly as slow intravenous (IV) infusion, can bind to mannose-6 phosphate (M6P) receptors on the cells surface through the M6P residues present on the oligosaccharide chains, and be targeted to lysosomes [15]. ERT was first developed for Gaucher disease in the ‘90s with optimal results [16]. Due to this success, to the lack of different therapies and to new orphan drugs legislation, ERTs for MPSs were developed and licensed from the beginning of the 2000s in the following order: MPS type I (2003), type VI (2005), type II (2006), type IVA (2014) and type VII (2017) [17,18,19,20,21,22]. Table 1 reports basic information on the diseases, enzyme defects and pharmacological and commercial names of the recombinant enzymes.

ERT in the present formulation has the relevant drawback that it does not cross the blood–brain barrier. Thus, at least for severe patients with progressive brain involvement, it really represents a “partial cure” [23]. Furthermore, similar issues are observed for all types of MPSs: there are tissues where the efficacy of the recombinant enzyme is limited by scarce penetration, like eyes, cartilage and bones, and tissues that cannot be repaired having an already-established damage at time of start of ERT [24]. A recent comparison between MPS I-Hurler patients treated with HSCT and others treated with only ERT showed impressive differences in hydrocephalus and cord-compression incidence in the two groups: 0% and 16%, respectively, for the HSCT-treated and 40% and 67%, respectively, for the ERT-only-treated patients [25]. The use of ERT in patients who develop severe cognitive decline might be questioned. At present the prevalent opinion among the scientific community is to start ERT for all those who do not have a more effective treatment in order to alleviate the somatic symptoms. The decision on whether to continue the treatment or not is made together with the family on the basis of periodic clinical evaluations [26]. The paper by Sato and Okuyama, in this Special Issue, illustrates the available information on clinical trials with novel enzyme replacement therapies for neuropathic MPSs [27].

In the early 2000s Gregory Grabowski wrote that our expectations about ERT’s efficacy in MPSs needed to be mitigated: due to the underlying different pathogenesis and pathology, MPSs could not be expected to improve like we had seen for Gaucher disease. [28]. Now, after about 15 years of experience with MPS I, II and VI, we can try to summarize ERT’s results. However, due both to the great variability of phenotypes, with different rates of progression, and to the different ages at starting treatment, the therapeutic effects in single individuals may be very different [24]. Additionally, these being complicated multisystem diseases, it is possible that the treatment addresses efficiently some but not all of the many signs and symptoms related to different organ/system dysfunction. In addition, due to young age or cognitive delay, not all patients are able to perform functional tests useful to quantify, in a reasonable time, the evolution of the phenotype and, indirectly, the effect of treatment [29]. Furthermore, we are still in search of reliable and validated biomarkers to monitor the response to therapy and to guide dose-optimization. Lastly, after phase III is completed and the drug is approved, since ERT is often the only treatment available, it would be unethical to perform randomized double-blind placebo-controlled studies. Hence most of the difficulties in evaluating the efficacy.

A review of journal articles was carried out on PubMed from 2000 to 2020: the subject headings were mucopolysaccharidosis (or MPS) I, II, IVA, VI, VII, enzyme replacement therapy, ERT, laronidase, Aldurazyme, idursulfase, Elaprase, idursulfase beta, Hunterase, elosulfase, Vimizim, galsulfase, Naglazyme, vestronidase alfa, Mepsevii. They were used alone or in combination. We also searched for each MPS type combined with the respective recombinant enzyme, both pharmacological and brand name and safety, antibodies, ear nose and throat (ENT), eyes, hearing, respiratory function, 6Minute Walking Test (6MWT), endurance, cartilage, bone, growth, siblings. In this review, we will summarize the present knowledge about the efficacy and effectiveness of ERT in the different tissues and the different outcomes in relation to age at starting treatment (siblings’ studies). We also reviewed available data about safety and development of anti-drug antibodies (ADAs) with its consequences. Table 2 summarizes the results of ERT by outcome parameters gathering together the different MPSs, in clinical trials and post-marketing studies.

## 2. Clinical Trials and Real-World Experience

Clinical trials are performed by definition in a limited time and, due to the need of obtaining quantifiable results, enroll only a certain kind of patients, who are able to perform well defined tasks such as for example 6MWT, or 12-Minute Walking Test (12MWT) or 3-Minute Stair Climbing (3MSC) and respiratory function tests. This implies that patients who are unable to perform these tests, like the very young ones or those with limited mobility or cognitive deficiencies, are excluded. Thus, the results of clinical trials only show what can be obtained in a limited time, in selected patients with defined characteristics. The real-world experience may be very different. Many evidences sometimes cannot be obtained because the patient is not collaborating [29] but, on the other hand, the long-term observation of the patients allows us to analyze other ERT effects like those on stature, heart, bones and cartilages, mobility and performance in activities of daily living (ADL), and quality of life (QoL). In the following paragraph (n.3), we will summarize the well-known data obtained from the clinical trials, and in paragraph 4 and its subheadings, we will report the outcomes of post-marketing studies divided by organ/system.

## 3. Data from the Clinical Trials about Efficacy of ERT

The phase III randomized, double-blind, placebo-controlled clinical trials are on the top of the evidence hierarchy in the evaluations of safety and efficacy because they compare the treatment with a placebo in a double-blind setting [24,108,109,110]. The clinical trials that have been performed for MPS’s ERTs have lasted from 24 to 52 weeks (MPS I-II-VI-IV-VII). Endurance with 6MWT (or 12MWT for galsulfase) and 3MSC for MPS VI and IV and respiratory function were chosen as primary and secondary endpoints [17,18,19,22]. The number of enrolled patients ranged from 12 (MPS VII-ultrarare disease) to 176 (MPS IVA). The patients were randomized 1:1 to receive a full dose of investigational product or placebo (MPS I and MPS VI) or randomized 1:1:1 to receive a full dose, half a dose and placebo in MPS II and MPS IVA trials. In the vestronidase alfa phase III trial for MPS VII, a novel blind start design was used to overcome the small sample size and the heterogeneity of the phenotypes of patients enrolled [21]. The results of all the trials showed a significant reduction of total GAGs excretion (keratan sulfate, KS, was tested instead in MPS IVA), reduction of liver size, significant improvement of endurance (6MWT, 12MWT and 3MSC), and improved joint mobility [17,18,19,21,22,30,31,32,33]. Mean 6MWT improved in a range from 20.8 (MPS VII) to 53 m (MPS VI) [21,111]. The evaluation of ERT efficacy on respiratory function was heterogeneous in the different trials. Forced vital capacity (FVC) and Apnea-Hypopnea Index (AHI) improved in the trials with laronidase (MPS I) and phase I/II elaprase (MPS II) [17,32]. In the phase II/III trials with elaprase (MPS II) and elosulfase (MPS IVA) FVC or maximal voluntary ventilation (MVV) were part of a tertiary composite endpoint considering both endurance tests and respiratory function [19,22]. FVC and forced expiratory volume in the first second (FEV1) did not improve in the MPS VI trial while MVV had a slight non-significant improvement [18]. Range of motion (ROM) improved in MPS I and II and in phase II trials of MPS VI [17,19,30,31], but not in the phase III MPS VI and MPS VII trials [18,21]. Reduced joint pain was reported for MPS VI, VII and IVA with limited ambulation [21,30,31,112]. In the phase III trial with elosulfase, Caregiver Assistance and Mobility Domain scores of MPS-Health Assessment Questionnaire (MPS-HAQ) were only slightly improved compared to controls after 24 weeks of ERT [98]. MPS-HAQ [22] is an adaptation for MPSs of the Health Assessment Questionnaire (HAQ) /Childhood Health Assessment Questionnaire (CHAQ) used for rheumatoid arthritis [113]. Idursulfase beta (Hunterase) underwent a phase I/II trial and is approved only in South Korea: the trial showed reduction of urinary GAGs (uGAGs) and improvement of 6MWT after 24 weeks with an acceptable safety profile [33]. Interestingly, in a preliminary open label study performed by Kakkis et al., an improvement of New York Heart Association (NYHA) functional classes and an amelioration of visual acuity were reported in 10 MPS I patients [59]. These two parameters were not considered in the subsequent trials with laronidase or the other ERTs.

The majority of the patients developed ADAs against the recombinant enzyme [17,18,19,21,22,114]. ADAs interference with the efficacy of the treatment is still debated [115].

The different drugs were licensed on the basis of the results of the phase III trials both in the United States and in Europe, being the first and only treatment for most of the patients. The questions arising about the long-term use in a heterogeneous (both for severity and different age at start of treatment) population are many and some of them are reported here: is the drug indicated for all patients or are there subgroups who will not reap enough benefit to balance the trouble of periodic infusions? – will the improvements seen in endurance and ROM be maintained during the years? – is this improvement powerful enough to improve the QoL? – will mucosal hypertrophy eventually disappear? – what about progressive tracheal malformations? will they improve or at least stabilize? – will bone abnormalities, including spine deformities, stabilize? – is urine GAGs measurement a reliable biomarker to allow monitoring of efficacy of treatment? –what about growth, on the long term? – and can we hope for improvements or stabilization of sensory functions? – could high titer neutralizing antibodies modify the response to ERT? The answers to these questions should come from the results of long-term post-marketing studies.

## 4. Real Life ERT

Post-marketing studies of ERTs suffer primarily from the lack of untreated patients or patients treated with different drugs to compare with. Notwithstanding this limitation, post-marketing studies are the only tool we have to gain knowledge on effectiveness in the long-term and in all kind of patients. Some of these studies are the phase III extension studies and involve the same patients previously enrolled in the Phase III trials [36,37,38,39,71,96,114]. The others are mostly observational studies conducted in a single center on a limited number of cases, or studies performed on data extracted from disease registries [29,34,37,40,41,42,43,44,47,48,49,50,51,52,70,74,77,84,85,97,116]. Disease registries are a helpful tool to collect data on rare diseases and there is now a huge number of disease registries available for many rare diseases. Often the registries are requested from the pharmaceutical companies as part of the approval for commercialization in order to obtain comprehensive data on safety and efficacy on the long-term and on a major number of patients. However, the multicenter and voluntary nature of the registries makes the contribution of data often incomplete and of variable quality [117].

### 4.1. GAGs and Other Possible Biomarkers

Total urine GAGs concentrations have been used in the Phase II/III trials as markers of the biochemical efficacy of ERT [17,18,19,21,22,30,31,33] and their level can be correlated with the different severity of phenotypes [118,119]. Their decrease is dramatic in the first 6–12 months of treatment and is sustained over the years tending to normalize in almost all patients [29,34,35,36,37,38,39,40,41,42,43,44,45,46,47,48,49,50,51,52]. However, blood and urine dermatan sulfate (DS) and heparan sulfate (HS) derived disaccharides, that are considered more suitable biomarkers for MPS I and II [120,121,122], were persistently elevated in long term ERT-treated MPS I and MPS II patients, and blood mono- and di-sulfated keratan sulfate were elevated in MPS IVA patients on ERT [122,123].

Numerous animal studies have shown that GAGs storage triggers a cascade of events leading to an increase of reactive species, disruption of redox metabolism with oxidative damage to molecules like lipids, proteins, DNA and RNA, and an increase in inflammatory citokines and apoptosis [124,125,126,127]. Alterations of these parameters were also observed in patients affected with MPSI, II, IVA and VI suggesting an important role in the pathophysiology of the disease [53,54,55,56,57,58]. They were only partially modified by the treatment with ERT [53,54,55,56,57,58]. It has been suggested that pro-inflammatory factors can be used as biomarkers for monitoring the efficacy of ERT. For this reason, anti-inflammatory/anti-oxidant drugs are proposed as an additive treatment to ERT [57,122].

In conclusion, biomarkers of tissue oxidative damage and inflammation do not normalize during ERT, although a partial protective role of long-term ERT against oxidative stress has been recognized [53,54,55]. This lack of full biochemical response to ERT might be partially related to antibodies production against the recombinant enzyme, to a suboptimal dosage of the enzyme, or to other factors currently not yet understood [121].

### 4.2. Organomegaly

Most long-term observational studies confirm a progressive reduction of spleen and liver size with a tendency to normalization [36,38,40,41,42,43,45,46,48,49,50,51,59,60,85]. Only some patients are reported with spleen and/or liver size stabilization [42,50,60]. This was already expected since preclinical studies on animals had shown a preferential bio-distribution of the drug to liver and spleen [128,129]. The reduction of liver size has probably a positive role in improving diaphragm excursions.

### 4.3. Ear Nose and Throat Manifestations

Upper airway related symptoms (macroglossia, enlarged adenoids and tonsils, reactive airway disease/asthma, recurrent otitis or sleep disturbances) are very frequent in all types of MPSs [2,130]. They are reported in 65% to 85% of patients in the MPS I registry [131]. Timpanostomy, adenoidectomy and tonsillectomy are reported in 51%, 49%, and 35%, respectively, of the patients enrolled in Hunter Outcome Survey (HOS), and 45%, 42% and 29%, respectively, of those surgical interventions were performed very early in life before the diagnosis of MPS II [132].

After the first data of improvement/stabilization of AHI (events/hour) found in a preliminary observational prospective study and in the MPS I patients originally enrolled in the phase II or III trials [36,45,59], only few other papers confirmed reduction of obstructive sleep apneas [46,61]. Muenzer et al. mentioned reduced obstructive sleep apneas (OSAs) among observed somatic improvements such as reduction in the frequency of respiratory infections, reduction of coarseness of facial features and improvement of ROM in severe Hunter patients, concluding that in 50 out of 66 patients, at least one of these improvements was seen after at least one year of ERT [26]. Reduction of respiratory infections in severe Hunter cases is also reported by Lampe et al. [34].

Dualibi et al. described improvement of ENT infections and rhinorrhea after 16–22 months of treatment with laronidase, while no improvement was seen in polysomnography and macroglossia, [62]. Pal et al. performed a multivariate analysis of sleep disordered breathing (SDB) in 61 MPS I patients, 41 of whom treated with HSCT and 20 with ERT. This analysis showed that, in patients on ERT, poor SDB outcome correlated with reduced substrate clearance, as demonstrated by a high urine dermatan sulfate: chondroitin sulfate (DS:CS) ratio, and high titers of inhibitory antibodies [133]. In general, HSCT treated patients had better results, but those on ERT with absent antibodies performed similarly to HSCT patients [133]. This study underlines the impact of ADAs on the outcome [128].

All the studies considering the size of adenoids and tonsils during ERT show no evidence of reduction of hypertrophy [40,49,50,62]. Low or very low evidence of improvement of ENT manifestation after ERT was also found in MPS I and MPS II patients treated in adult age [134,135].

Recently Keilmann et al. quantified the mucosal alterations of the hypopharynx and larynx in 15 MPS patients on ERT and found no trend of improvement [63]. Similarly, Pal et al. showed increased lysosomal compartment size, increased GAGs storage and increased extracellular matrix (ECM) deposition in the adenotonsillar tissues of seven MPS children treated with ERT for a long time [64].

These observations suggest that, once established, substrate deposition is not cleared by ERT, in accordance with all the previous clinical observations of a lack of size reduction of adenoids and tonsils and need of adenotonsillectomy while on ERT [50,62,73]. Summarizing, ERT possibly reduces the number of infections and sleep apneas, but no effect on macroglossia, and mucosal and adenoids-tonsils hypertrophy is demonstrated.

### 4.4. Respiratory Function

Tracheobronchomalacia is a frequent finding in MPSs, mainly in MPS II and IVA [65,136,137,138,139], substantially contributing to an increased risk while undergoing anesthesiological procedures. It is due both to an intrinsically malacic trachea and to external compression by mediastinal deposition of GAGs or a tortuous brachiocephalic artery [65,66,67,68,136,137,139], in the context of disproportionate growth of the thoracic cage. During the years of observation of ERT-treated MPS patients, it has become clear that ERT is not effective neither on tracheal cartilage deformities nor in clearing the extrinsic mediastinal storage [65,66,67,68]. Nevertheless, improvements were seen in spirometric evaluations (FEV1, FVC and MVV) according to most of the long-term studies [36,37,38,43,49,69,70,71,72]. Muenzer et al. found improvement of FVC only in patients younger than 18 [38]. FEV1 and FVC were stabilized from baseline in severe and less severe MPS VI patients after 8.2 and 6.2 years, respectively [70]. Cardiopulmonary exercise tests performed on a small group of Morquio A patients enrolled in the MOR-008 study suggested that ERT improves aerobic efficiency, as demonstrated by an increased performance of work at reduced metabolic demand [140]. Some studies showed instead a stabilization or mild decline, less relevant if compared to untreated patients [60,73]. Kenth et al. examined, over the course of 10 years, 16 subjects with MPS IVA, 13 of which were on ERT. They tested, every 6–6.6 months, overnight oximetry, FEV1 and FVC, and calculated their percent predicted value and their ratio. During the study period they saw a general reduction of spirometry values, with a delayed decline in the ERT group and a mild non-significant increase over time of median percent arterial oxygen saturation in patients on ERT [73]. Kiliç et al. studied FEV1 and FVC in 4 MPS VI patients before and after starting ERT [60]. Two patients were within normal limits with no changes due to ERT; the other two patients had restrictive and obstructive signs and after ERT one was unchanged and the other worsened [60].

In conclusion, although many of the causes of decreased respiratory function in MPSs (large tongue, hypertrophy of adenoids and tonsil, excessively long U-shaped trachea and/or tracheal narrowing, restricted thoracic cage and short stature) are not modified by ERT, an improvement of pulmonary function, albeit partial, is obtained with ERT.

### 4.5. Heart

Cardiac valve thickening, left ventricular hypertrophy, conduction abnormalities, and coronary artery disease are major features of heart disease in MPSs [141].

While it is generally acknowledged that long term ERT reduces or at least stabilizes left ventricular mass index and interventricular septal hypertrophy, and improves ejection fraction in MPS I, II, IVA and VI [40,41,42,46,51,74,75,76,77,78], valve abnormalities causing regurgitation or stenosis are not reduced by ERT [34,36,37,40,41,44,46,47,51,59,74,75,76,77,78,79]. Only few papers found stabilization or worsening of heart hypertrophy in MPS VI and II patients [34,37,47]. The effect of ERT on coronary arteries has not been systematically explored but there are reports of coronary arteries involvement in ERT treated patients who suffered of sudden or unexpected death with findings of myocardial infarction or coronary artery stenosis at autopsy [83,84]. An important observation is that made in siblings treated at different ages: the oldest sibling being treated as symptomatic and the youngest at an earlier age when still asymptomatic [142,143]. ERT administered within the first months of life appears to prevent valve involvement or at least substantially attenuate the course of the valve damage process [141,142,143]. This observation is supported by similar results obtained in MPS I mice treated with ERT from birth [144].

### 4.6. Vessels

Carotid intima-media thickness (cIMT) and arterial thickness are non-invasive measures of sub-clinical atherosclerosis and were studied in MPS I patients treated with ERT or previously treated with HSCT compared to healthy controls. An increase of thickness was found in all the MPS patients at all ages [81]. Aortic root dilatation was recently evaluated in two different papers that showed a prevalence ≥ 35% in all MPS types with the highest occurrence in MPS IVA and MPS VI patients (>70%) [80,82]. Other studies underline the vulnerability of the cardiovascular system in MPS and especially in MPS IVA [145,146]. To date, it seems that neither ERT nor HSCT have an impact on these vessels’ abnormalities [121,122,123].

### 4.7. Hearing

Hearing loss, conductive, sensorineural and mixed, is very frequent in MPSs, has early onset and is progressive [48,86,147,148,149]. No data on the impact of ERT on hearing function is available in the clinical trials nor in the open label extension studies [17,18,19,22,36,37,38]. Hearing function was recently evaluated in 124 MPS patients of all types, most of whom were on ERT: no effects of ERT on hearing changes were found [86]. The impact of ERT has also been evaluated in small case series with inconclusive results. Some papers have reported improvement in a number of MPS VI patients after years of ERT [42,47,49] while progressive worsening in MPS I and MPS VI patients on ERT has also been described [45,60,85]. In conclusion, no clear effect of ERT on hearing loss has been demonstrated.

### 4.8. Eyes

No effects of ERT on corneal clouding was reported in the first 10 MPS I patients treated with laronidase, while most subjects showed decreased photophobia or conjunctival irritation, and in one patient improved visual acuity was observed [59]. Two reviews suggest that available data seem to indicate a stabilization or slower progression of corneal clouding with ERT, with extreme variability from one patient to another, probably due to different severity and different age at starting ERT [150,151]. Scarpa et al. found a worsening of eye symptoms in two of nine patients with MPS VI after a short duration ERT, while stabilization of corneal clouding was reported for other MPS I and VI patients on ERT [87,88,89]. More recently, Sornalingham et al. studied patients treated with HSCT or ERT and, although no improvement was evident in the ERT-treated patients, they found an association between severity of corneal clouding and urinary levels of biomarkers, namely DS/CS in MPS I and VI and KS/CS in MPS IVA [90]. A recent review evaluates all English language literature found in PubMed from 1981 to 2018 and concludes that, while HSCT stabilizes or improves corneal clouding, ERT has no effect on the cornea [152]. The lack of effect on the eye is usually attributed to the presence of the retina–brain barrier and to the avascular nature of the cornea. However, an animal study suggests that this barrier might be crossed through increasing the dosage: MPS I dogs treated with high dose of ERT (1.58-2 mg/kg/week) showed significantly less GAGs accumulation in the cornea compared to no-treatment or low-dose (0.58 mg/kg/week). No-treatment and low-dose groups did not show any difference in GAGs accumulation [153].

There are very few data regarding other aspects of ocular involvement in MPS. However, there are reports of improved visual acuity in one MPS I patient and in another 4 with MPS VII [21,59] and an isolated report of reversed papilledema in an MPS VI patient on galsufase [154].

### 4.9. Joints

Joint stiffness is one of the main problems in four of the five ERT-treatable MPSs. MPS IVA is conversely the only MPS with joint laxity and hypermobility, leading very early to subluxations and osteoarthritis in adulthood [155]. Restricted joints limit the normal ADL such as dressing up, buttoning up jackets, tying laces, combing hair, picking up coins, putting a hat on the head and so on. Passive ROM measurement is easy to perform with a goniometer assessing degrees and can be performed in all patients, without limitations of age or developmental quotient. Being a partially subjective measure, it is important that it is always performed by the same professional [91]. Joint restriction is progressively worsening with age [91,156]. There is quite many data on ROM in long-term extension open-label trials and in observational post-marketing studies, unanimously showing a sustained improvement over the years, albeit partial, or stabilization, in at least 50% of the patients. However, significant changes of ROM from baseline were only obtained for shoulders [34,36,38,40,41,42,49,51,59,91,92,93]. Clarke et al. reported that the patients with the most severe impairment at baseline were those who showed the best improvement [36]. Two studies on MPS I and MPS II identified the amelioration of ROM as the main cause of improvements in ADL and consequently in QoL [34,45]. Improvement in mobility was seen in 6 of 12 MPS VII patients treated with vestronidase alfa and 6 out of 12 patients showed fine motor issues and self-care improvement [21,157]. Conversely, Cox-Brinkman et al. did not find any ROM improvement in MPS I after one year of treatment with laronidase [94] and Guarany et al. did not see any difference between treated and not-treated patients with different MPSs [95]. In conclusion, response of joint restriction to ERT is probably variable, depending on age and individual compromise.

### 4.10. Endurance and Mobility Issues

Endurance, tested with 6MWT or 12MWT or 3MSC had a prevalent place in the clinical trials, these tests being quantifiable and easy to perform. The only problem is that they need both cognitively adequate and collaborating patients. This excludes cognitively normal children below 5–6 years of age, cognitively retarded patients, and those who are wheel-chair bound.

6MWT, 12MWT and 3MSC provide a comprehensive measure of the cardiovascular and the respiratory apparatus as well as of the osteo-articular system function [158,159].

Improvement or stabilization of 6MWT have been reported for MPS I, II, IV and VI after 1–3 years of treatment [36,38,39,40,41,42,43,51,52,96,97]. For MPS II and VI there are data of longer observations for 4–7 years showing a mean increase around 65 m or more [37,40,42,70]. 3MSC (used to test MPS IV and VI patients) and 12MWT (used only for MPS VI) gave similar results [39,42,52,70,96,97]. Berger et al. showed very limited improvement of 6MWT and 3MSC after one year in relatively well-doing Morquio A patients enrolled in the MOR-008 trial. This result was associated with improved cardiopulmonary function (see above) and the authors suppose that it might be attributed to a ceiling effect limited by other conditions like orthopedic abnormalities [140].

Taken together, these data show an undoubted impact of ERT on the natural history outcome of mobility issues in MPSs. MPSs are progressively worsening diseases and finding an improvement from baseline after many years of treatment ought to be considered a real success [33,80,112,113]. However, it has been objected that it should be verified whether the changes in 6MWT represent or not the “minimal clinical important difference” (MCID) for patients. There are no studies evaluating the MCID for the 6MWT in MPSs or in other LSDs, but the results obtained for MPS IVA were compared to the MCIDs determined in patients with respiratory, cardiovascular, or musculoskeletal disease (from +7 to +9 % change): the placebo-adjusted improvement in the elosulfase trial was 14.9% after 24 weeks and 20.7% after 2 years of treatment, versus a reduction of 6.9% in comparable untreated patients [160].

### 4.11. Impact of ERT on Patient Reported Outcome

Self-assessed questionnaires inquiring about pain, fatigue, ADL, QoL, psychological health, help physicians to understand the impact of disease progression or therapies in the single patient. The subjectively-filled-out answers represent the Patient Reported Outcome (PRO) and help in finding, together with the patient, what particular deficit or limitation is considered more disturbing by the single individual and needs to be treated first. For example, hearing loss may be more important for someone than carpal tunnel syndrome or vice versa, depending on temperament, job, interests. PRO measures have been included in several clinical trials as efficacy end points. CHAQ/HAQ or MPS-HAQ questionnaires have been used more frequently [161].

Improvement in CHAQ/HAQ Disability Index has been reported for MPSI and MPS II after 1–6 years of ERT [36,38,45,91]. Parini et al. found improvements in mobility, self-care and independence domains scores of MPS-HAQ on the long term (9 years) only in cognitively competent patients [40]. Worsening of performance in ADL in severe Hunter patients has also been reported by Tanjuakio et al. [162].

MPS IVA individuals showed sustained improvement in all domains of the MPS-HAQ (mobility, self-care and independence) after 2 years of ERT [100]. Six Spanish patients were assessed with EuroQol- 5 Dimensions-5 Levels (EQ-5D-5L), Visual Analog Score (VAS) after 8 months of ERT: three showed stabilization and three showed improvement of the score [97]. Lin et al. reported improved dexterity, reduced joint pain and stiffness after 6.2–11 years ERT in MPS VI patients while Giugliani et al. found no changes in 55 MPS VI after long term ERT [37,42].

Joint pain is frequent: reported by 27–40% of the MPS I, II, VI patients participating in a nationwide survey in the Netherlands [163]. Some studies using PRO questionnaires found a partial response of pain to ERT [31,36,164].

### 4.12. Bones and Growth

GAGs deposition in bone cartilage produces progressive damage to the growth plate, with the consequence of incomplete ossification and short stature. This characteristic is part of skeletal dysplasia which includes prominent forehead, kyphoscoliosis, platyspondyly and other vertebral abnormalities, pectus carinatum, tracheal deformities, hip dysplasia and genu valgum [165]. Bone dysplasia in severe forms is recognizable at birth and has neither been reversed nor stabilized in patients who were already symptomatic when started ERT [166,167]. Furthermore, early peri-transplant ERT in MPS I Hurler did not modify the development of carpal tunnel syndrome compared to untreated patients [168].

All types of MPSs experience abnormal growth with increased length at birth and decline of growth velocity beginning from 1 to 5–6 years of age [99,101,165,169,170,171,172,173]. A complete or partial lack of pubertal growth spurt is also common in all MPS types [99,101,106,169,171,174]. In the first years of life, short height is more pronounced in MPS IVA, MPS I and MPS VI, but the final height is always shorter than healthy individuals in all MPSs, even in MPS III, the least affected type for growth [171]. In each type the more severe growth impairment is seen in the more severe patients [172,175].

The effect of ERT on growth on the long term has been studied in MPS I, MPS II, MPS IVA, MPS VI [37,45,47,70,101,102,103,104,105,106,107]. Many studies report limited improvement of growth after starting ERT [37,45,70,101,102,103,104]. A few long-term studies did not find any efficacy of ERT on growth, even when started at a young age [40,105,106,107]. Doherty et al. have recently showed that the growth of 128 Morquio A patients on ERT did not differ from that of untreated patients [106].

In general, some studies show a limited effect of ERT on growth in patients who had started treatment before puberty or at least below 15 years [37,45,70,101,102,103,104]. Furthermore, patients showing a growth delay before commencing ERT, did not obtain a tangible improvement [101,102,103,104].

### 4.13. ERT in Siblings

A series of impressive clinical observations in siblings treated at different ages showed that ERT is reasonably able to modify the clinical course of the disease if administered at an early age [142,143,176,177,178,179,180,181]. These case studies are only observations and not blind placebo-control studies, but they are relevant enough because they all go in the same direction. All the youngest really had a better evolution than the oldest from a multisystem point of view. Different evolution in the youngest siblings involved development of facial bones, joint stiffness, other skeletal deformities and growth, organomegaly and heart valves [79,80,145,146,147,148,149]. In two families with MPS VI, joint mobility was preserved in the youngest sibling but progressive bone deformities and corneal clouding had developed also in the youngest sibling [176,177]. All together, these cases underline the importance of early treatment. Animal studies on MPS mice are consistent with these findings [144,182,183]. According to these data on newborn MPS mice and human MPS siblings, there is no doubt that newborn screening is needed for all the MPS that can benefit from an etiologic treatment.

### 4.14. Effects of Discontinuation of ERT

The effects of ERT discontinuation could be evaluated retrospectively in very few cases of MPS I and MPS II patients described in the literature [184,185,186]. A severe early worsening was observed after discontinuation, with relapse of organomegaly, reduced endurance and reduced percent predicted FVC [184,185,186].

### 4.15. Peri- and Post-Transplant ERT

ERT has become a routine supportive/adjuvant treatment in the peri-transplant period in MPS I Hurler patients [187,188]. It is generally agreed that ERT is started at diagnosis and continued until full engraftment [185,187,189]. ERT is well tolerated and no increased risk was found of either graft versus host disease or failure of engraftment [187]. It was also effective in improving pre-transplant clinical conditions and notably in reverting cardiomyopathy and severe heart failure in a few months in patients who later underwent HSCT [188,190,191]. Very early administration at 34 days of life allowed the prevention of coarse facial features in a girl who underwent HSCT at 9 months, but the vertebral abnormalities present at birth did not change at 3 years post-transplantation; furthermore, at the same age, metacarpal bones and phalanges abnormalities, absent at birth, became clearly recognizable. These observations confirm that bone deformities are a very difficult target in the treatment of MPS [167].

The efficacy of post-transplant ERT, years after successful HSCT, has been anecdotally described in one MPS I and one MPS VI patient [192,193]. Recently it has been the object of a study, reported in two different papers, on 10 MPS I patients starting ERT at least two years after HSCT and treated for two years [194,195]. Remarkably, the authors observed a reduction in uGAGs and improvement in 6MWT only in the seven patients who did not develop high-titer neutralizing antibodies (NAbs). The three patients with high-titer NAbs had no improvement of 6MWT and an increase of uGAGs. Thus, the apparently limited advantages of post-transplant ERT in those who did not develop high NAbs need to be balanced with the possible interference of NAbs, since the increased uGAGs probably reflect the inhibition of the uptake both of the exogenous and endogenous enzyme [194,195].

## 5. Safety

Overall, ERTs for MPSs are generally well tolerated and have an acceptable safety profile. Most infusion associated reactions (IARs) were mild to moderate and included rash, urticaria, angio-edema, bronchoconstriction, rhinitis, pyrexia, nausea, vomiting. They were easily resolved by interrupting or slowing down the rate of infusion and/or by the administration of anti-histamines, antipyretics and/ or corticosteroids. Most patients who experienced IARs received and tolerated subsequent infusions. IARs were reported in around 35–70% MPS patients on ERT, but only 1–2% were considered severe [18,19,21,22,36,41,43,45,46,47,114,196,197,198,199]. Very rare serious adverse reactions, including life-threatening anaphylaxis have been reported, both in clinical studies and in postmarking experience, even after several dozen infusions of ERT [21,36,200,201,202]. However, IARs episodes were shown to decrease progressively with time during treatment in the extension studies [36,38].

To minimize the risk of IARs, pretreatment is recommended 30–60 min prior to the start of the infusion and may include antihistamines, antipyretics, and/or steroids as well as careful consideration of the patient’s clinical status prior to administration [203].

In the case of recurrent IARs, with failure of pre-medication to prevent hypersensitivity reactions, desensitization is indicated. This is a process inducing a state of temporary tolerance through sequential exposure to increasing doses of the drug tricking the immune system into accepting it. Desensitization should be performed in an appropriate setting with the support of an allergologist and /or immunologist, due to the possible reactions. It is usually indicated in patients who have an IgE-mediated reaction appearing within a few hours after the start of infusion [204]. Effective desensitization has been reported in patients treated with laronidase, idursulfase, galsulfase and elosulfase [202,205,206,207,208].

There is little information about the safety of ERT during pregnancy and lactation. In the very few cases reported, no adverse effects of ERT on pregnancy and on breastfed infants have been observed in any of treated patients with MPSI, MPS IV, and MPS VI [209,210].

## 6. Immunogenicity

In MPSs, similarly to other LSDs, recombinant enzymes infused intravenously frequently lead to the production of ADAs and, notably, of NAbs, that are able to impair the biological activity of the protein. The enzymes are perceived as non-self proteins by the immune system, they are taken up by antigen-presenting cells, then processed and presented to T helper specific cells. Helper T-cell signals activate proliferation of antigen-specific B cells that differentiate into memory B cells and into antibodies secreting plasma cells. The effects of immune response may be complex, and ADAs might interfere with the desired biological effects of the therapeutic enzyme through different mechanisms: altered enzyme targeting, increased enzyme turnover, inhibition of the enzyme uptake by the cells, inhibition of catalytic site. The majority of ERT-treated MPS patients produce ADAs in the first months of treatment but a longer exposure time is required to develop NAbs [17,18,19,30,31,32,36,41,49,114,199,201,211,212].

The overall IgG levels in young children with MPSI were reported to be higher and the time to seroconversion to be shorter compared to older patients, but with no noticeable differences in terms of IARs [46]. Moreover, among MPS II patients, younger age is a predictor of immunogenicity [37].

A progressive decline of ADAs over time was also reported suggesting the development of a natural immune tolerance in MPSI [17,36,44] and has been seen at a certain extent also in MPS II and IVA [38,211].

ADAs are mainly IgG, and much more rarely IgE. The link between IgE and the development of hypersensitivity reactions is not uniform in all forms of MPSs, since positive IgE ADAs were found in Hunter patients with anaphylaxis, while in Morquio A patients, no apparent correlation between IgE ADAs and hypersensitivity reactions was noticed [200,201]. Inevitably, the residual production of the endogenous enzyme affects the propensity of the patient to generate ADAs and NAbs. This has been well shown in patients affected by Pompe disease, in which a clear relationship between humoral immune response and both residual protein production and clinical outcomes has been proved [213].

For MPSs instead, more than 15 years after the first approved ERT for MPSs, the question of whether ADAs have a clinical impact and to what extent this may be the case, is not yet fully understood [115]. There is a report of association between genotype and immune response in young children with severe MPS II phenotype: patients with nonsense or frameshift mutations were more likely to develop antibodies and showed a reduced uGAGs response compared to patients with missense mutations [199]. No clear relationship has been reported in MPS I and II between exposure to higher ADAs and risk of adverse events or suboptimal clinical outcome, except in one case of MPSI whose ADAs inhibited cellular uptake of enzyme by more than 80% [44,196,199].

In more than one study, ADA-positive patients are associated to higher uGAGs levels and in some cases to poorer outcome [18,19,32,36,133,196,214,215]. No impact was seen for vestronidase [21]. The presence of NAbs has been associated with a less robust efficacy response to treatment also in terms of liver size in MPS II [215], while no apparent correlation between NAbs titer and clinical outcome has been observed in Morquio patients [114,201,211].

A more complex immune response to ERT, including both humoral and T cells response, cannot be ruled out, as recently suggested by Squeri et al., who studied MPS I mice and found the activation of an innate response inducing anti-IDUA CD8+ T cells in concomitance with ERT [216].

Attempts to prevent ADA responses to ERT were seldom described. An immune tolerance induction regimen based on cyclosporin A and azathioprine was ineffective in patients with MPS IH as well as monotherapy with methotrexate [217,218]. Another immune tolerance induction regimen was used in a 4-year-old MPS II boy, with sustained high ADAs titer and limited clinical efficacy of idursulfase treatment. Over 18 months, therapy with atumumab, bortezomib, methotrexate, short-term dexamethasone, and IVIG resulted in a significant reduction of neutralizing anti-idursulfase IgG titer and a moderate reduction in uGAG levels compared to baseline, while modest clinical improvement was observed [219]. A prophylactic immune modulation treatment, has been employed in a patient with Morquio A with a large deletion causing minimal or no protein production, therefore predicting a greater risk of developing antibodies to ERT [220]. This approach led to a greater reduction of urine KS compared with clinical trial patients, but the long-term clinical significance remains to be determined [220]. Recently a new approach with a combination of non-depleting anti-CD4 and -CD8 monoclonal antibodies in a mouse model was able to induce partial or complete immune tolerance to laronidase [218]. A recent case report shows the successful response to a non-immunosuppressive and non-cytotoxic immune tolerance regimen consisting of intravenous immune globulin and frequent small infusions of idursulfase (twice a day reaching 1 mg/kg weekly) administered to a young boy with MPS II. In this case NAbs levels significantly correlated with uGAGs [221].

Concluding, the effect of ADAs in MPSs is difficult to evaluate probably due to the slowly- progressive course of these diseases and the partial efficacy of the treatment. We still have limited data on the real impact of immunogenicity on effectiveness of ERT for MPSs, and the question whether the presence of ADAs has clinical significance or not remains unsolved. ADA testing is not always easy to perform in the clinical setting and the time lapse to obtain the results may be long. Monitoring ADAs is crucial in the post-approval setting and should be part of the routine management of each patient on ERT.

## 7. Conclusions

In this review we discussed the relevant available data on efficacy of ERT in MPSs. Taken together, the results are very similar for the different MPSs. By examining each outcome measure, we could conclude that a clear therapeutic effect of the presently available ERTs is seen only for few outcomes. For others the results are partially controversial and give a scenario of limited efficacy restricted to few organs/systems (Table 2). GAGs decrease and reduction of organomegaly are well-recognized effects of ERTs: from the clinical point of view these two effects are marginal but demonstrate a sustained biological effect. Clinically more important is the quite universal reduction of left ventricular mass index and of septum hypertrophy, with ejection fraction improvement [40,41,46,49,51,52,74,75,77]. AHI and OSAs, when evaluated, seem to be reduced by ERT, but it is clear that macroglossia and adeno-tonsils hypertrophy are not modified during long-term treatment [36,45,46,49,50,61,62,63,64]. Although only few cases are reported, it appears that ERTs do not act on deformities of trachea, bronchi and vessels [65,66,67,68,81,82,83,84]. In the first years after ERT availability it seemed that it could have a “stabilization” role on hearing loss and corneal clouding but unfortunately the different articles do not agree on this point and the final impression is that hearing and eyes are among the difficult-to-reach targets for ERT [42,47,59,60,85,86,87,88,89,90]. ROM and endurance tests are the most frequently used tests in the trials and have been tested in the extension phase III studies and in many observational studies. The better ROM results showed significant improvement only for the shoulders with non-significant ameliorations for the other joints [34,36,38,40,41,42,49,51,59,91,92,93]. The endurance tests have always improved during the pivotal trials and the improvement was maintained during years of observation. For example, the data obtained after approximately 2 years of ERT in MPS IVA show a sustained improvement which is much more impressive compared to the data of Morquio A Clinical Assessment Program (the natural history study with a protocol parallel to that of the pivotal trial), which showed a worsening in a 2-year period [96].

Growth is another field where results are quite discouraging. Improvement, when it was seen, was in general mild and clinically not significant. Bones are a difficult target for ERT both because the damage is starting very early in fetal life and due to scarce penetration. However, the studies of siblings showed that the earlier-treated siblings had a better growth [142,143,176,181]. This is a demonstration that ERTs have the potential to impact bones, but it is possible only if the treatment is started at a very early age.

ADAs role should be better explored and the effects of inhibition of ADAs production should be tested in clinical studies. There is also a need for good biomarkers, able to help in monitoring the evolution of the disease. The data obtained from siblings clearly shows that early treatment is better and this is a strong argument for developing neonatal screening for all treatable MPSs. The use of traditional ERTs in combination with other treatments or new ERTs with different properties or new drugs with different approaches, to be used alone or in combination, will help in improving prognosis of MPS patients over the next years.

## Figures and Tables

**Table 1:** Classification of mucopolysaccharidoses (MPSs) with types, syndromes’ names, phenotype Mendelian Inheritance in Man (MIM) number (#), deficient enzymes with their Enzyme Commission (E.C.) classification, gene symbol, affected glycosaminoglycans (GAGs) (DS = dermatan sulfate, HS = heparan sulfate, KS = keratan sulfate, CS = chondroitin sulfate) inheritance (AR= autosomal recessive; XL = X-linked), names of recombinant enzymes and their commercial name. Data obtained from Online Mendelian Inheritance in Man^®^ (OMIM^®^) https://www.ncbi.nlm.nih.gov/omim. Accessed on 7 April 2020.

Type	Syndrome	Phenotype MIM Number (#)	Deficient Enzyme (EC Classification)	Gene Symbol	Affected GAGs	Inheritance	Recombinant Enzyme	Brand Name
MPSIH H/S S	Hurler Hurler/Scheie Scheie	607014- 607015- 607016	Alpha-L-iduronidase (3.2.1.76)	IDUA	DS,HS	AR	Laronidase	Aldurazyme^®^
MPSII	Hunter	309900	Iduronate 2-Sulfatase (3.1.6.13)	IDS	DS,HS	XL	Idursulfase alfa Idursulfase beta	Elaprase^®^ Hunterase^®^
MPSIVA	Morquio A	253000	Galactosamine-6-sulfatase (3.1.6.4)	GALNS	KS,CS	AR	Elosulfase	Vimizim^®^
MPSVI	Maroteax-Lamy	253200	Arylsulfatase B (3.1.6.12)	ARSB	DS	AR	Galsulfase	Naglazyme^®^
MPSVII	Sly	253220	Beta-glucuronidase (3.2.1.31)	GUSB	DS,HS,CS	AR	Vestronidase	Mepsevii^®^

**Table 2:** Summary of results by outcome parameters in clinical trials and post-marketing studies of enzyme replacement therapy (ERT) in MPSs. The main findings of outcome parameters are reported summarizing data of all types of MPSs together.

Outcome Parameters	Characteristics of the Study	Type MPS	Age (Years)	Number of Patients	Main Findings	References
uGAGs	Phase II/III Clinical Trials	I, II, IV, VI, VII	5–57	440	Significant reduction after 6 months	[17,18,19,21,22,30,31,32,33]
	Extension studies/observational	I, II, VI	0.17–58	558	Persistent reduction after years	[29,34,35,36,37,38,39,40,41,42,43,44,45,46,47,48,49,50,51,52]
OXIDATIVE STRESS, PRO-INFLAMMATORY BIOMARKERS	Research studies in human subjects on ERT	I, II, IV	1–36	60	Partial improvement or no effect on lipid peroxidation, protein oxidative damage, DNA damage and increased pro-inflammatory cytokines	[53,54,55,56,57,58]
ORGANOMEGALY	Phase II/III clinical trials	I, II, VI	5–45	264	decreased	[17,18,19,21,30,31,32,33]
	Extension studies/observational	I, II, VI	0.17–58	384	Decreased-normalized	[36,38,40,41,43,45,46,48,49,51,59]
	Observational	II, VI	1.25–29.5	49	Only some decreased, the others unchanged	[42,50,60]
OSAs/AHI	Phase II/III trials	I, II	6–43	47	Decreased AHI	[17,32]
	Extension studies/observational	I, II	0.5–43	82	Decreased AHI	[36,45,46,59,61]
ADENO-TONSILS HYPERTROPHY	Extension studies/observational	I, II, IIIA, IVA, VI,	1–27	96	No improvement	[40,49,50,62,63,64]
TRACHEOBRONCHOMALACIA/MEDIASTINAL STORAGE	Observational	II, IVA, VI	12–50	8	No improvement	[65,66,67,68]
SPIROMETRY TESTS (FEV1, FVC, FEV1%, MVV)	Phase II/III	I, II, IVA	5–57	339	improvement	[17,19,22,31,32]
	Phase II/III	VI	5–29	39	No improvement	[18]
	Extension/observational	I, II, IVA, VI	2–58	715	Stabilization or improvement	[36,37,38,41,43,49,69,70,71,72]
	Observational	IVA, VI	1.25–43	17	Stabilization or gradual decline	[60,73]
HEART HYPERTROPHY	Extension/observational	I, II, IVA, VI	0.5–53.9	177	Improvement or stabilization	[40,41,42,46,51,74,75,76,77,78]
	Extension/observational	II, VI	1.5–37	70	Stabilization or worsening	[34,37,47]
HEART VALVES	Extension/observational	I, II, IVA, VI	0.5–53.9	336	Stabilization or worsening	[34,36,37,40,41,44,46,47,51,59,74,75,76,77,78,79]
VESSELS	Observational	I, II, IVA, VI	3.4–27.8	54	No apparent efficacy on vessels abnormalities	[80,81,82,83,84]
HEARING	observational	VI	0.17–58	31	Stabilized or improved	[42,47,49]
	Observational	I, II, IVA, VI	1.25–29.5	174	Stabilized or worsened	[45,60,85,86]
EYES- CORNEAL CLOUDING	observational	I	5–22	8	No apparent efficacy	[59]
	Observational	I, IVA, VI	4–44	38	Stabilization or worsening	[87,88,89,90]
JOINT RESTRICTION (MEASURED WITH PASSIVE ROM)	Phase II/III trials	I, II, VI	5–43	154	Mild improvement	[17,19,30,31,33]
	Phase II/III trials	VI, VII	5–35	64	No improvement	[18,21]
	observational	I, II, VI	0.2–53.9	257	Stabilized or improved	[34,36,38,40,41,42,49,51,59,91,92,93]
	observational	I, VI	8–31	12	No improvement	[94,95]
ENDURANCE (6MWT, 12MWT, 3MSC)	Phase II/III trials	I, II, IVA, VI, VII	5–57	437	Improvement	[17,18,19,21,22,30,31,33]
	observational	I, II, IVA, VI	0.7–58	577	Improvement maintained after 3 or more years	[36,37,38,39,40,41,42,43,51,52,70,96,97]
HEALTH RELATED QUALITY OF LIFE QUESTIONNAIRES (MAINLY MPS-HAQ, CHAQ/HAQ)	Phase II/III trials	IVA	5–57.4	176	Mild improvement	[98]
	observational	I, II, IVA, VI	2.3–57.4	415	Improved or stable	[36,37,38,42,45,91,97,99,100]
GROWTH	observational	I, II, IVA, VI	0–58	628	Limited improvement before 15 years of age	[37,45,70,101,102,103,104]
	observational	I, II	0.4–23.1	171	No improvement	[40,105,106,107]

Notes: uGAGs = urinary glycosaminoglycans, OSAs = obstructive sleep apneas, AHI = apnea hypopnea index, FEV1 = forced expiratory volume in the first second, FEV1% = percent predicted FEV1, FVC = forced vital capacity, MVV = maximal voluntary ventilation, ROM = range of motion, 6MWT = 6 min walking test, 12MWT = 12 min walking test, 3MSC = 3 min stair climbing, MPS-HAQ = MPS- health assessment questionnaire, CHAQ = childhood health assessment questionnaire, HAQ = health assessment questionnaire.
